# AAV‐mediated *Gpm6b* expression supports hair cell reprogramming

**DOI:** 10.1111/cpr.13620

**Published:** 2024-02-24

**Authors:** Qiuhan Sun, Liyan Zhang, Tian Chen, Nianci Li, Fangzhi Tan, Xingliang Gu, Yinyi Zhou, Ziyu Zhang, Yicheng Lu, Jie Lu, Xiaoyun Qian, Bing Guan, Jieyu Qi, Fanglei Ye, Renjie Chai

**Affiliations:** ^1^ State Key Laboratory of Digital Medical Engineering, Department of Otolaryngology Head and Neck Surgery, Zhongda Hospital, School of Life Sciences and Technology, Advanced Institute for Life and Health, Jiangsu Province High‐Tech Key Laboratory for Bio‐Medical Research Southeast University Nanjing China; ^2^ Northern Jiangsu People's Hospital Affiliated to Yangzhou University/Clinical Medical College Yangzhou University Yangzhou China; ^3^ Department of Otolaryngology‐Head and Neck Surgery, the Affiliated Drum Tower Hospital of Nanjing University Medical School Jiangsu Provincial Key Medical Discipline(Laboratory) Nanjing China; ^4^ Department of Otology The First Affiliated Hospital of Zhengzhou University Zhengzhou Henan China; ^5^ Department of Otolaryngology Head and Neck Surgery, Sichuan Provincial People's Hospital University of Electronic Science and Technology of China Chengdu China; ^6^ Co‐Innovation Center of Neuroregeneration Nantong University Nantong China; ^7^ Institute for Stem Cells and Regeneration Chinese Academy of Science Beijing China; ^8^ Southeast University Shenzhen Research Institute Shenzhen China

## Abstract

Irreversible damage to hair cells (HCs) in the cochlea leads to hearing loss. Cochlear supporting cells (SCs) in the murine cochlea have the potential to differentiate into HCs. Neuron membrane glycoprotein M6B (*Gpm6b*) as a four‐transmembrane protein is a potential regulator of HC regeneration according to our previous research. In this study, we found that AAV‐ie‐mediated *Gpm6b* overexpression promoted SC‐derived organoid expansion. Enhanced *Gpm6b* prevented the normal decrease in SC plasticity as the cochlea develops by supporting cells re‐entry cell cycle and facilitating the SC‐to‐HC transformation. Also, overexpression of *Gpm6b* in the organ of Corti through the round window membrane injection facilitated the trans‐differentiation of Lgr5^+^ SCs into HCs. In conclusion, our results suggest that *Gpm6b* overexpression promotes HC regeneration and highlights a promising target for hearing repair using the inner ear stem cells combined with AAV.

## Graphical abstract

In this work, Sun et al. investigated the effect of up‐regulation of *Gpm6b* on the hair cell differentiation of progenitors using Adeno‐associated virus‐inner ear (AAV‐ie)–mediated gene overexpression. In this study, Sun et al. found that *Gpm6b* overexpression could promote supporting cell proliferation and hair cell differentiation through organoid culture experiments in vivo and viral injection in neonatal mice.

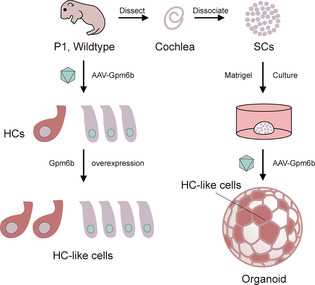

## 1 INTRODUCTION

Hearing loss is a common disease worldwide and has a profound impact on patients' communication and language acquisition.^1^ There are many causes of deafness including ageing, ototoxic drugs, overstimulation, infection and genetic factors.^2^ Hearing recovery is very difficult due to damaged HCs in the mature mammalian cochlea are unable to spontaneously regenerate.^3^ Currently, the ideal treatment for sensorineural deafness would be to regenerate HCs from cochlear progenitor cells such as SCs to repair the structure and function recovery of the cochlea and restore hearing. Therefore, exploring effective methods for HC regeneration is the key question of current hearing research.^4^

The sensory precursor cells proliferation and the HCs regeneration in the inner ear are regulated by various signalling pathways such as Wnt, Notch, Bmp/Smad, IGF (Insulin‐like growth factor) and FGF (fibroblast growth factor).^5^ Simultaneously Wnt signalling pathway activation and Notch signalling pathway suppression can stimulate the hair cell differentiation of progenitors during the development of the cochlear epithelia, resulting in the production of ectopic HCs.^6^ Inhibition of the Bmp signalling pathway increases HC regeneration after streptomycin injury,^7^ and the Wnt signalling pathway can interact extensively with multiple signalling molecules such as TGF‐β and BMP to allow overlapping signalling pathways to specify cell fates,^8^ thus playing a crucial role in regulating inner ear HC regeneration. However, it has not been possible to regenerate new HCs comparable to native HCs in mammals. Therefore, it is crucial to screen new factors that promote HC regeneration.

Glycoprotein GPM6B‐a four‐transmembrane protein belonging to the lipid‐protein family of integrated membrane proteins‐was originally defined as a structural protein of the myelin sheath of the central nervous system.^9^ The GPM6B protein is generally expressed throughout the brain and expressed in neurons, oligodendrocytes and astrocytes,^10^, ^11^ and it is involved in the differentiation of terminally differentiated cells in various mammalian tissues. It also plays an important role in neuronal myelination and neuronal differentiation, which is essential for the correct extension and guidance of axons in the corpus callosum.^12^ By stimulating the TGF‐β‐Smad2/3 signalling pathway, the GPM6B protein can induce the differentiation of vascular wall smooth muscle cells.^13^ As a multifunctional cytokine, TGF‐β can regulate the morphogenesis of various cells as well as the processes of cell proliferation and differentiation.^14^ TGF‐β1 is expressed in the inner ear and has been shown to enhance the protective effect of GDNF (Glial cell line‐derived neurotrophic factor) against ototoxicity caused by aminoglycoside‐induced HC loss.^15^ In addition, Smad‐2 and Smad‐3 promote chondrogenesis in the developing middle ear capsule.^13^ Therefore, we hypothesised that the GPM6B protein has a function in the regeneration of inner ear HCs.

The inner ear is structurally and anatomically independent from other organs, facilitating the direct delivery of exogenous genes, and the fluid‐filled environment allows the diffusion of therapeutic genes. AAV‐ie, screened by our team previously, as a new and safe AAV vector can efficiently infect mostly inner ear SCs in mice, and has been shown to effectively achieve the overexpression of Atoh1 in SCs and induce HC regeneration.^16^ Moreover, SCs and HCs are derived from the same pool of progenitor cells. Therefore, in this research, we used the self‐designed AAV‐ie to deliver the *Gpm6b* into the cochlear progenitor cells through the round window membrane injection. We found that the overexpression of *Gpm6b* accelerated the SCs proliferation and HC differentiation in the cochlear organoids. RNA‐seq results showed that HC regeneration resulting from *Gpm6b* overexpression was related to Wnt, Hippo, and other signalling pathways. A similar phenomenon was observed in vivo, where *Gpm6b* overexpression slightly promoted SC division and HC differentiation, while lineage tracing indicated that regenerating HCs were partially derived from Lgr5^+^ SCs.

## 2 RESULTS

### 2.1 AAV‐*Gpm6b* promoted SCs proliferation in the cochlear organoids

**FIGURE 1:**
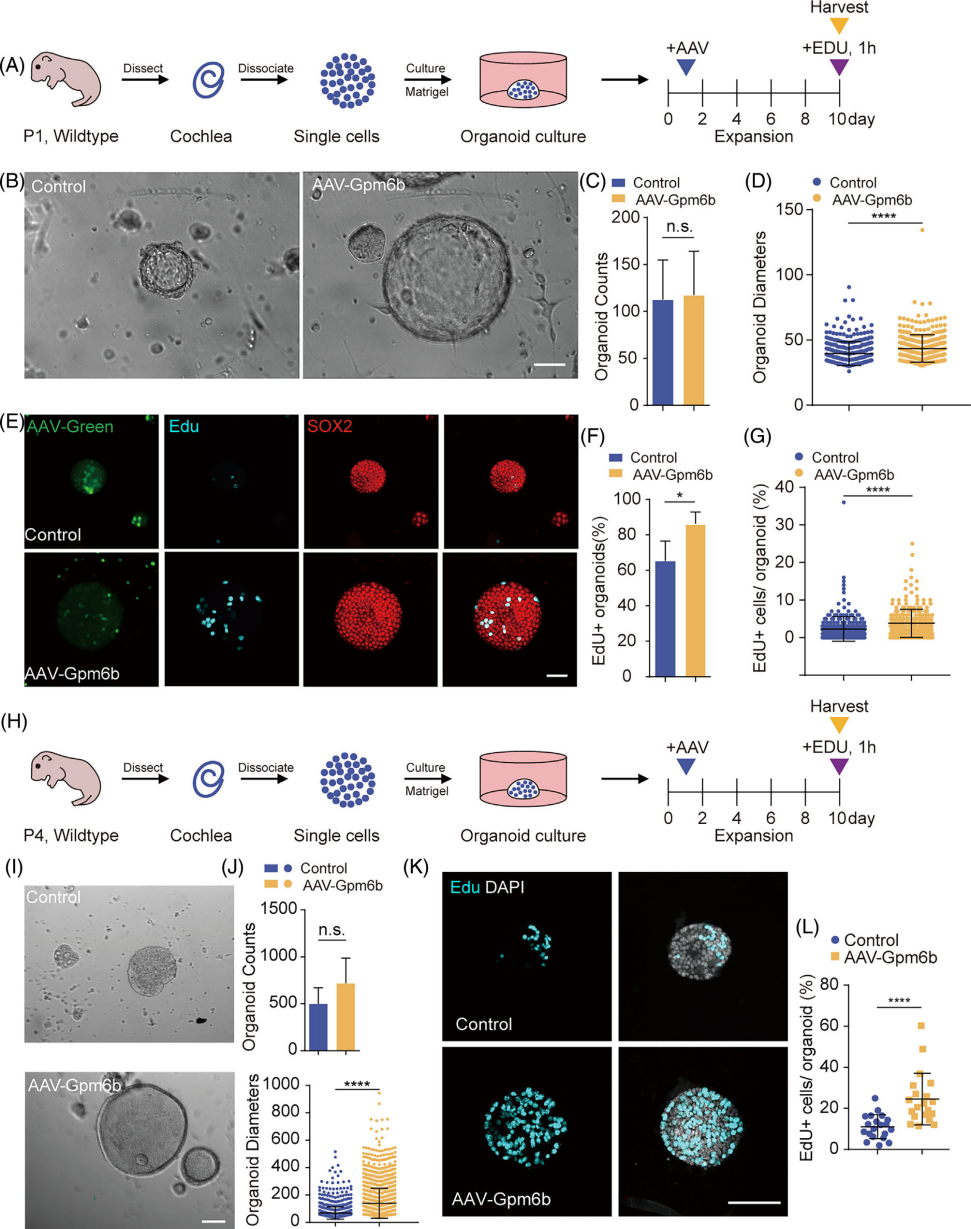
*Gpm6b* overexpression promoted the proliferation of cochlear organoids. (A) Experimental design of the expansion culture of SCs in three‐dimensional in vitro assay. Given a single EdU treatment for 1 h on day 10. (B) Bright‐field images of cochlear organoids after expansion with the administration of AAV‐mNeonGreen (Control) and AAV‐*Gpm6b*, respectively. Scale bar: 50 μm. (C and D) The number (C) and diameter (D) of the organoids in (B). (E) Immunofluorescence images of the organoids in (B). Sox2 (red) marks SCs. EdU (cyan) marks proliferating cells. AAV‐mNeonGreen (green) marks cells transduced by AAVs. Scale bar: 50 μm. (F and G) The ratio of EdU^+^ organoids (F) and the ratio of EdU^+^ cells per organoid (G) in (E). (H–L) Similar analyses to (A to G) of cochlear organoids from P4 mice. Scale bars: 100 μm. AAV dose: 2 × 10^10^ GCs per well. Data are displayed as the mean with SEM. The value of *p* was calculated by Student's *t‐*test. *p < 0.05; ****p < 0.0001; n.s., no significance.

It has been shown that SCs have the potential to differentiate into HCs.^17^, ^18^ SCs serve as HC progenitors in the postnatal mice cochlea, so we explored the effect of *Gpm6b* overexpression on SC proliferation and differentiation into HCs in vitro. The cochlear organoid is an excellent model for studying supporting cell plasticity. Control virus AAV‐mNeonGreen (a green fluorescent protein) and the experimental virus AAV‐*Gpm6b*‐mNeonGreen were added in the medium during the organoid culture (Figure 1A,B). We evaluated the diameter and number of organoids, which can be used as criteria to evaluate the proliferation ability of stem cells.^19^ Our results showed that the organoids significantly increased in diameter but did not change in number after *Gpm6b* overexpression compared to the control group (Figure 1C,D). Before collecting the samples, EdU was added for 1 h in order to label the proliferating cells. The immunofluorescence images showed that the percentage of EdU+ SCs in each organoid and the proportion of EdU+ organoids increased significantly after *Gpm6b* overexpression compared to the control group (Figure 1E–G). Studies have shown that the plasticity of mouse SCs declines rapidly after birth.^19^ In order to explore whether *Gpm6b* overexpression has a positive effect on the proliferation ability of cochlear SCs in the aged mice, a similar three‐dimensional organoid culture was performed on P4 WT mice and AAV‐Gpm6b was introduced in the organoid (Figure 1H,I). Similarly, after *Gpm6b* overexpression the diameter of the organoids increased significantly, without a changeable number of organoids (Figure 1J). Moreover, the immunofluorescence staining of the organoids showed that the percentage of EdU+ SCs also increased significantly in each organoid after *Gpm6b* overexpression (Figure 1K,L). In conclusion, *Gpm6b* overexpression can facilitate SC proliferation in the culture organoid.

### 2.2 AAV‐*Gpm6b* stimulated HC regeneration in cochlear organoids

**FIGURE 2:**
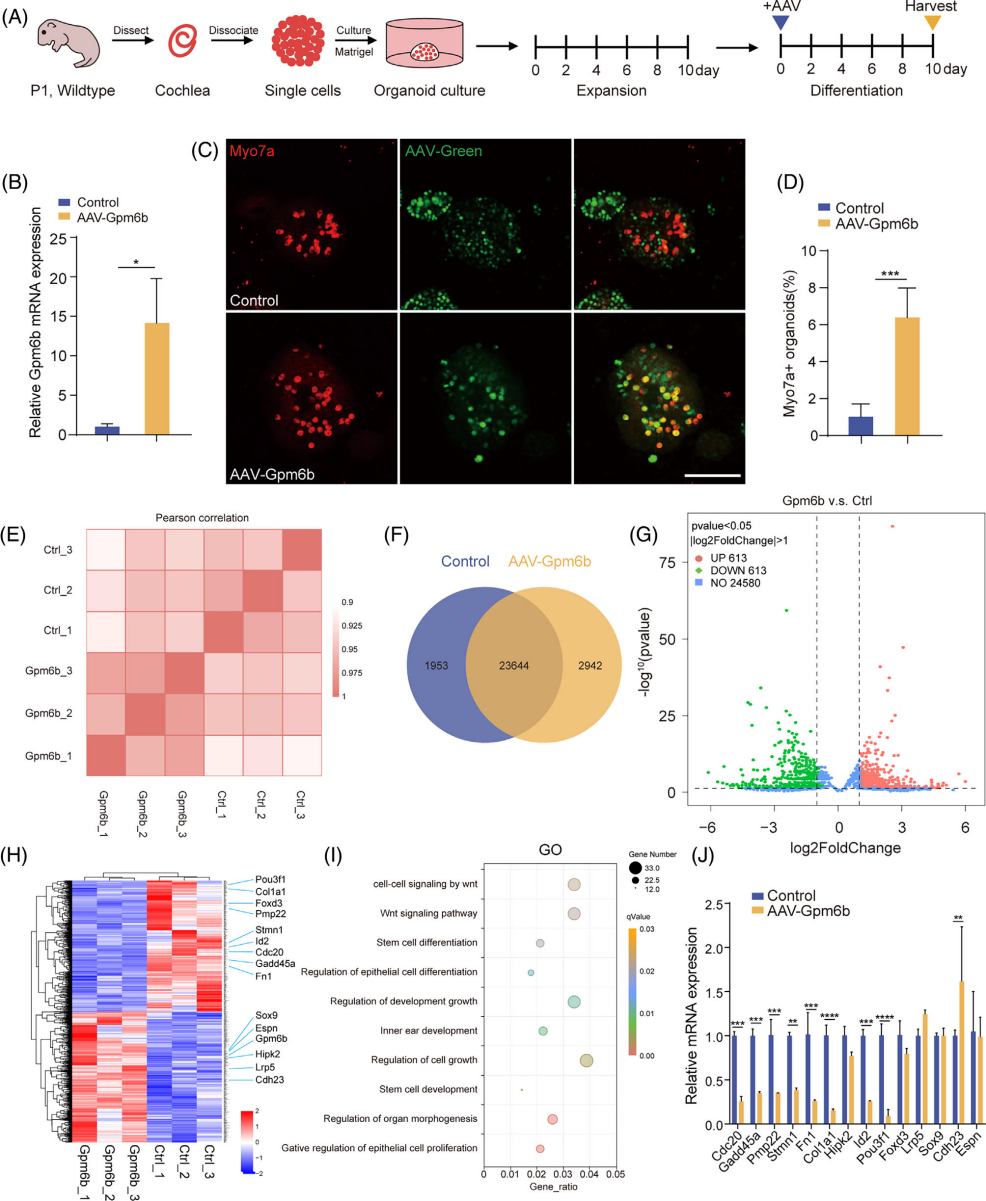
*Gpm6b* overexpression promoted HC regeneration in cochlear organoids. (A) Experimental design of the differentiation culture of cochlear organoids in the three‐dimensional in vitro assay. AAV dose: 2 × 10^10^ GCs per well. (B) Relative mRNA expression of *Gpm6b* in the cochlear organoids. (C) Immunofluorescence images of the organoids in (A). Scale bar: 100 μm. (D) The Myo7a^+^ organoids ratio was calculated in (C). (E–I) RNA sequencing data from cochlear organoids was added with control and AAV‐*Gpm6b*, respectively. (E) Correlation analysis between control and AAV‐*Gpm6b*–transduced organoids. (F) Venn diagram analysis of differentially expressed genes. (G) The volcano plot shows the overall profile of differentially expressed genes. Red/green dots indicate up/down‐regulated genes, respectively. Genes with no statistically significant expression difference are shown as blue dots. **|**log2FoldChange**|** > 1 and *p*‐value < 0.05 were the cutoffs for differentially expressed genes. (H) Heatmap of the differentially expressed genes. (I) GO terms significantly enriched in differentially expressed genes in *Gpm6b*‐overexpressing organoids. (J) The differentially expressed genes identified in (H) were verified by qPCR. Data are displayed as the mean with SEM. The value of *p* was calculated by Student's *t‐*test. **p < 0.01; ***p < 0.001; n.s. refers to no significance.

Next, we explored the differentiation ability of organoids derived from P1 mouse cochlear SCs after *Gpm6b* overexpression (Figure 2A). On the first day of differentiation culture, the AAV‐mNeonGreen or AAV‐Gpm6b was added, and immunofluorescence staining indicated that the percentage of Myo7a + organoids remarkably increased after *Gpm6b* overexpression (Figure 2B–D), suggesting that *Gpm6b* overexpression can promote the transdifferentiation of SCs into HCs.

To determine how *Gpm6b* overexpression improves the capacity of SCs to transdifferentiate into HCs, we analysed the transcriptomes of the control and *Gpm6b*‐overexpressing organoids using the RNA sequencing (Figure 2A). Pearson correlation between samples indicated that the samples between control and AAV‐*Gpm6b* transduced organoids had high similarity (Figure 2E). A Venn diagram showed that 1953 and 2942 genes were enriched in the control and *Gpm6b* group, respectively, and 23,644 genes were co‐expressed in both groups (Figure 2F). The gene expression changes induced by *Gpm6b* overexpression are shown in the volcano plot and heat map (|log2FoldChange| > 2.0, *p*‐value < 0.05) (Figure 2G,H). After *Gpm6b* overexpression, 613 genes were down‐regulated (blue) and 613 genes were up‐regulated (red) compared with the control group. To identify the signalling pathways and biological processes that might be altered by *Gpm6b* overexpression, gene ontology (GO) enrichment analysis was performed. Differentially expressed genes were highly enriched in functional categories such as the Wnt signalling pathway, stem cell differentiation and inner ear development (Figure 2I). We performed qPCR verification of the differentially expressed genes obtained by the RNA sequencing, and it showed that genes related to the cell cycle (*Cdc20*, *Gadd45a*, *Pmp22* and *Stmn1*), transcription factors (*Pou3f1* and *Foxd3*), the EGF signalling pathway (*Fn1* and *Col1a1*) and the TGF‐β signalling pathway (*Id2*) were down‐regulated, while genes associated with the Wnt signalling pathway (*Lrp5*), the Hippo signalling pathway (*Hipk2*) and the cochlear hair cell (*Cdh23* and *Espn*) were all up‐regulated (Figure 2J). Taken together, the above results suggested that *Gpm6b* overexpression can facilitate the transdifferentiation of SCs into HCs via multiple signalling pathways.

### 2.3 AAV‐*Gpm6b* promoted HC regeneration in the murine cochlea

**FIGURE 3:**
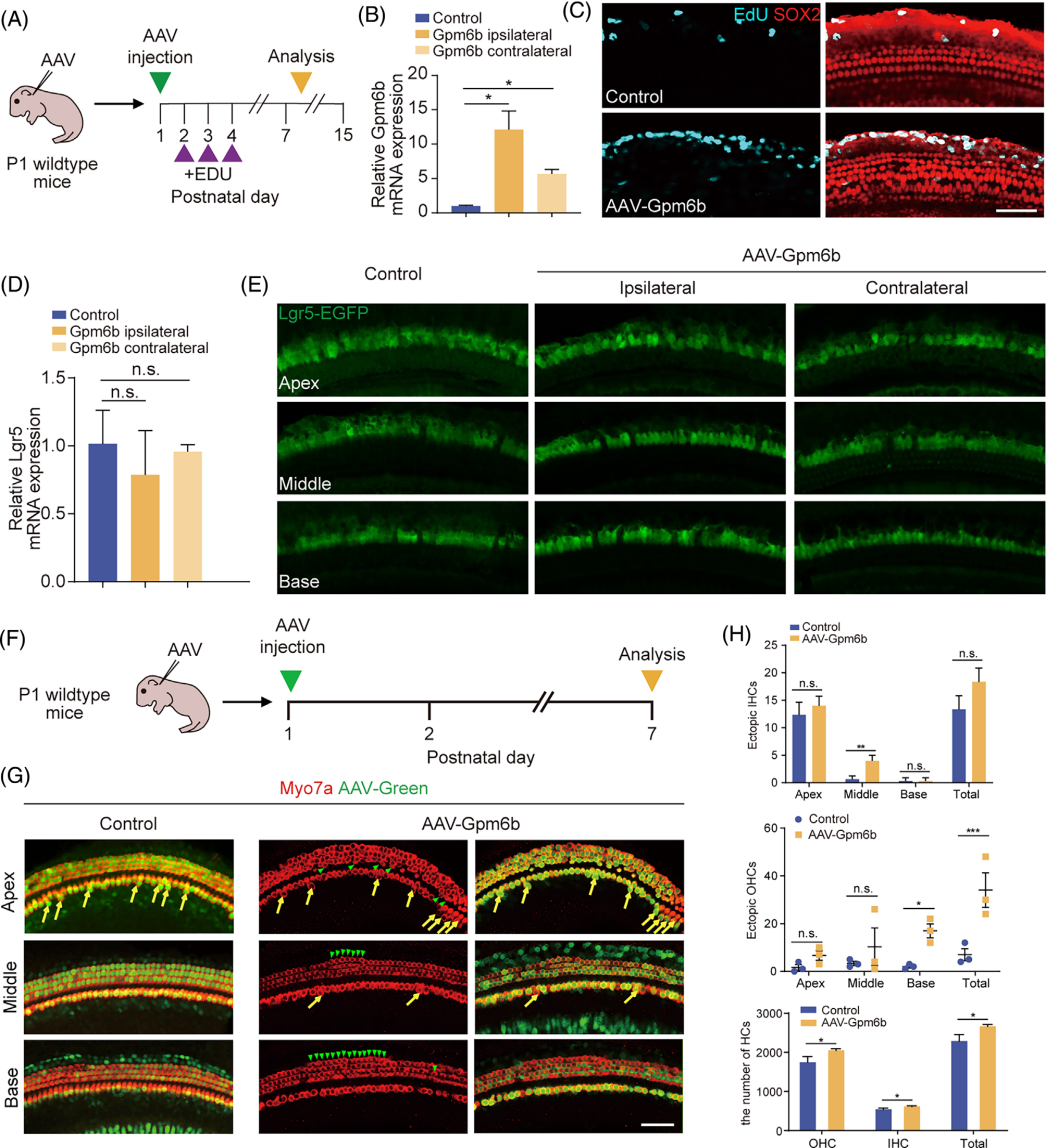
*Gpm6b* overexpression promoted hair cell regeneration in vivo. (A) Experimental design. (B) mRNA expression level of *Gpm6b* in P15 cochleae after the injection of AAV‐*Gpm6b*. (C) EdU immunostaining after *Gpm6b* overexpression in cochlear. EdU (cyan) marks proliferating cells. Sox2 (red) marks the SCs. Scale bar: 50 μm. (D) mRNA expression level of *Lgr5* in P15 cochleae after the injection of AAV‐*Gpm6b*. (E) Immunofluorescence images of the AAV‐transduced cochleae of Lgr5‐EGFP mice. Lgr5‐EGFP (green) marks the Lgr5^+^ cells. (F) Experimental design. Tamoxifen was injected intraperitoneally into P1 wildtype mice. The AAVs were injected through the round window membrane, and the cochleae were collected at P7. (G) Immunofluorescence images of cochlear epithelia infected by control and AAV‐*Gpm6b*, respectively. Ectopic HCs are marked by yellow arrows (IHC area) and green arrows (OHC area). Scale bar: 50 μm. (H) The number of ectopic IHCs, ectopic OHCs, and total HCs in (B). AAV dose: 2.8 × 10^10^ GCs per cochlea. Data are displayed as the mean with SEM. The value of *p* was calculated by Student's *t‐*test. *p < 0.05; **p < 0.01; ***p < 0.001; n.s. refers to no significance.

We further investigated the influence of *Gpm6b* overexpression on SC proliferation and HC regeneration in vivo. AAV‐mNeonGreen and AAV‐*Gpm6b* viruses were injected into P1 WT neonatal mice through the round window membrane, respectively. And, EdU was injected subcutaneously into P2–P4 mice to label the proliferative SCs. The cochleae were dissected at P7 and immunofluorescence staining was performed (Figure 3A). No EdU^+^ SCs (Sox2^+^/EdU^+^ cells) was detected in the control or *Gpm6b* group (Figure 3C). It was known that Lgr5 is a receptor of the Wnt signalling pathway and is considered as a marker of SCs in cochlea.^20^, ^21^ Lgr5 expression decreases gradually during SC development. Therefore, we obtained the cochleae of P15 Lgr5‐EGFP mice injected with AAV‐mNeonGreen and AAV‐*Gpm6b* viruses respectively, and our qPCR and immunofluorescence results showed that *Gpm6b* overexpression had no effect on the expression of Lgr5 in cochlea (Figure 3B,D,E). These results indicated that *Gpm6b* overexpression had no effect on the proliferation of SCs in vivo. We then explored the influence of *Gpm6b* on the SC‐to‐HC trans‐differentiation process in vivo. In P1 WT mice, the control and *Gpm6b* viruses were injected through the round window membrane, and the cochleae were dissected at P7 for immunofluorescence staining (Figure 3F,G). The data indicated the number of ectopic inner HCs (IHCs) in the cochlea increased after *Gpm6b* overexpression. The number of ectopic IHCs in the middle turn was remarkably different from the control group, while in the apical turn and basal turn were not significantly different. In addition, the number of ectopic outer HCs (OHCs) increased remarkably. The number of total OHCs and HCs were also increased significantly (Figure 3H). These results suggested that *Gpm6b* overexpression may promote the transdifferentiation of SCs into HCs in the cochlea.

### 2.4 AAV‐*Gpm6b* facilitated Lgr5^+^ SC‐to‐HC transformation in murine cochlea

**FIGURE 4:**
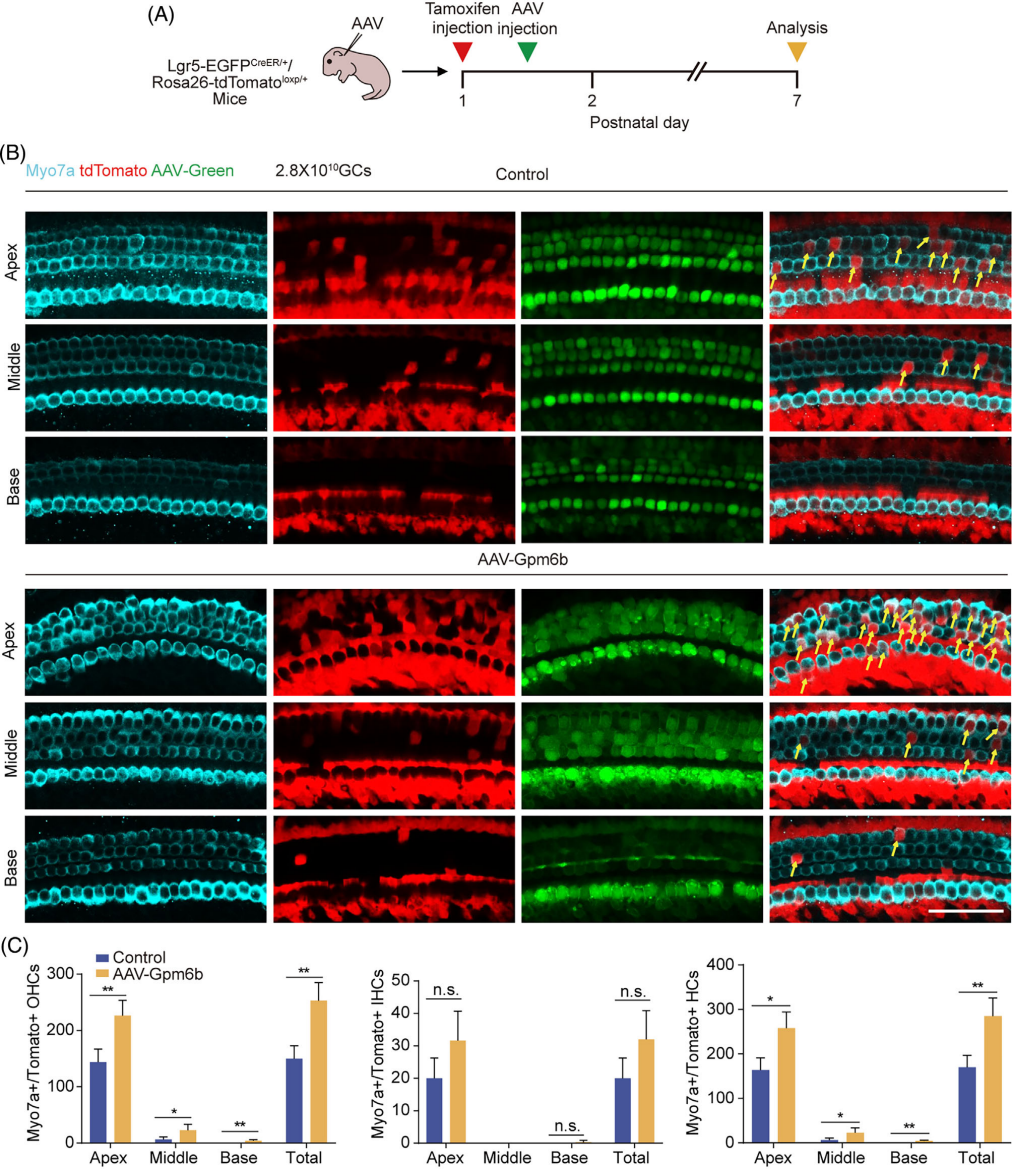
*Gpm6b* overexpression facilitated the trans‐differentiation of Lgr5^+^ progenitors into HCs. (A) Experimental design. P1 Lgr5‐EGFP^CreER/+^/Rosa26‐tdTomato^loxp/+^ mice were injected with tamoxifen intraperitoneally. AAVs were injected 0.5 days later through the round window membrane, and the cochleae were collected at P7. (B) Immunofluorescence of AAV‐transduced cochleae. Scale bar: 50 μm. (C) The number of Myo7a^+^/Tomato^+^ OHCs, IHCs and HCs per cochlea. AAV dose: 2.8 × 10^10^ GCs per cochlea. Data are displayed as the mean with SEM. The value of *p* was calculated by Student's *t‐*test. *p < 0.05; **p < 0.01; n.s. refers to no significance.

Lgr5^+^ SCs are usually considered as the HC progenitors, which can differentiate into HCs.^22^, ^23^ Therefore, we used Lgr5‐EGFP^CreER/+^/Rosa26‐tdTomato^loxp/+^ mice to explore the source of regenerated HCs. AAV‐ie can infect Lgr5^+^ SCs.^16^ Via round window membrane, we injected AAV‐mNeonGreen and AAV‐*Gpm6b* viruses in P1.5 mice, in which the Cre enzyme was activated by tamoxifen at P1. At P7, the cochleae were dissected for immunofluorescence staining (Figure 4A,B). Myo7a^+^/Tomato^+^ IHCs, Myo7a^+^/Tomato^+^ OHCs and Myo7a^+^/Tomato^+^ HCs were found in the control group and *Gpm6b* overexpression group. After *Gpm6b* overexpression, Tomato‐labelled OHC, not IHC, was increased significantly (Figure 4C). These results indicated that *Gpm6b* overexpression promoted the regeneration of cochlear HCs and that these regenerated HCs were derived from Lgr5^+^ SCs.

## 3 DISCUSSION

In previous studies, our team compared differentially expressed genes of different transcriptomes of SCs including Lgr5^+^ SCs in the apical/basal turn, the Lgr5^+^/Lgr5^−^ SCs, and Lgr5^+^ SCs from neomycin‐treated and non‐treated cochleae, and intersection analysis showed that *Gpm6b* expression changed significantly,^24^, ^25^, ^26^ suggesting that *Gpm6b* might be play a role in the regulation of HC regeneration. In this study, we used inner ear organoid culture in vitro and lineage tracing of neonatal inner ear HCs to show that the overexpression of *Gpm6b* improved the monopotency of SCs and promoted the transdifferentiation of SCs into HCs. This study identified the new gene that directs HC regeneration via AAV‐mediated gene delivery, thus providing new therapeutic targets for the prevention and recovery of auditory dysfunction caused by HC degeneration.

Due to the lack of spontaneous regeneration ability of inner ear HCs in mammals, there is currently no effective means to completely recover the loss of inner ear HCs.^27^ Studies have shown that SCs can be used as progenitor cells of the inner ear, which can divide or transdifferentiate to regenerate HCs,^28^ and some signalling pathways or transcription factors are associated with HC regeneration, for example, Atoh1, Wnt and Notch signalling pathways.^29^ Many other genes and related pathways have also been shown to play important roles in HC regeneration. These pathways can communicate with each other to influence the SC proliferation or HC regeneration.^30^ Gpm6b was originally identified as a structural protein in the nervous system of vertebrate embryos.^10^ And it is also expressed in peripheral tissues such as the smooth muscle, heart, skeletal muscle, liver, spleen and kidney.^31^ Gpm6b is a pleiotropic molecule with different function. It is involved in cellular housekeeping functions such as membrane transport and intercellular communication in the central nervous system.^32^ While, there are no studies of *Gpm6b* in the auditory system. In this study, we found that AAV‐*Gpm6b* could promote more SCs differentiation into HCs under physiological conditions compared with the control in mice, and perhaps our study will provide a theoretical basis for future human auditory system research about *Gpm6b*.

RNA‐seq data showed cycle genes (*Cdc20*, *Gadd45a*, *Pmp22* and *Stmn1*), transcription factors (*Pou3f1* and *Foxd3*), signalling pathway genes, including Wnt (*Lrp5*), Hippo (*Hipk2*), TGF‐β (*Id2*) and EGF (*Fn1* and *Col1a1*), and cochlea‐related genes (*Cdh23*, and *Espn*) were differentially expressed in the two groups of organoids. Several genes above have been confirmed to be associated with inner ear development and HC regeneration in mice. For example, the downstream target *Id2* is downregulated in AAV‐*Gpm6b*‐transduced TGF‐β signalling, which engages in cross‐talk with the classical Notch, Wnt and Hippo signalling pathways regulating the differentiation of HC progenitors, and this negatively regulates HC generation during the development of inner ear.^8^, ^33^ *Gpm6b* can also stimulate TGF‐β‐Smad2/3‐related signalling pathways that participate in the differentiation of osteoblasts and smooth muscle cells.^13^ The TGF‐β signalling pathway is also associated with the regulation of inner ear sensory epithelial cell status.^34^ Moreover, it has been shown that TGF‐β1 is expressed in the inner ear and enhance the protective effect of GDNF (Glial cell line‐derived neurotrophic factor) against ototoxicity caused by aminoglycoside‐induced HC loss.^15^ RNA‐seq results showed that TGF‐β1 was up‐regulated after *Gpm6b* overexpression, which might have a protective effect on HCs. Wnt signalling pathway activation can enhance the expansion of Lgr5^+^ cochlear progenitor cells,^22^ and Smad protein, a key mediator of the TGF‐β signalling pathway, can also respond to Wnt signalling. Notch signalling inhibition can cause Lgr5^+^ progenitor cells to re‐enter the cell cycle and transdifferentiate into HCs,^35^ and the TGF‐β‐Smad and Notch signals can affect the regenerative ability of stem cells through antagonistic interactions.^36^ Furthermore, the Hippo signalling pathway as well as YAP‐mediated overexpression of Lin28a can activate the Wnt pathway to facilitate HC regeneration,^37^ and YAP has been shown to be associated with Smad2/3/4 protein complexes and to determine their intracellular localisation.^38^ Thus, there are complex interactions between the Notch, Wnt and Hippo signalling pathways, indicating that regulation of multiple pathways may be more effective in promoting HC regeneration than regulating individual pathways. TGF‐β signalling plays an important role in these signalling networks, so it might be possible to use TGF‐β as a connector to further explore the signalling pathway crosstalk including *Gpm6b* to promote inner ear HC regeneration. Therefore, multiple signals upon *Gpm6b* overexpression may synergistically promote inner ear organoid proliferation and differentiation into HCs.

Cadherin 23 (Cdh23) is an important constituent of the HC tip link in the organ of Corti. Mutations in *Cdh23* are associated with age‐related hearing loss (AHL).^39^ Similarly, *Espn* has a crucial role in the structural maintenance and development of HC stereocilium.^40^ RNA seq. results showed that *Cdh23* and *Espn* were upregulated when *Gpm6b* overexpression. Therefore, *Gpm6b* overexpression can promote the relative HC genes high expression and HC differentiation. HCs act as specialised neurons, so we speculate that *Gpm6b* may also have a role in neuron regeneration and protection.

We also found reduced expression of cell cycle factor *Cdc20* in the AAV‐*Gpm6b*‐induced differentiation of inner ear organoids. *Cdc20* is involved in proteolysis mediated by ubiquitin and is highly expressed in proliferating cells and mediates the co‐regulation of MAD mitotic spindle checkpoint proteins, with the anaphase promoting complex (APC).^41^ *Cdc20* is expressed in the inner ear. At embryonic day 12.5, *Cdc20* is expressed throughout the cochlear duct epithelium, and it is significantly decreased in the prosensory region at embryonic day 14.5, which corresponds with the exit of prosensory cells from the cell cycle. Conditional knockdown of *Cdc20* in the anterior sensory region induces four or more rows of OHCs at the apex of the cochlea and leads to a reduction in the length of the cochlear canal.^42^ It is unclear whether *Cdc20* can control the generation of SCs and HCs by regulating the proliferation and differentiation of progenitor cells, but it is known that Cdc20‐APC is needed in presynaptic axonal differentiation of cerebellum postmitotic neurons.^43^ The relationship between *Cdc20* and HCs requires further investigation. In addition, many other differentially expressed genes, such as *Gadd45a*, *Pmp22*, *Stmn1*, *Fn1*, *Col1a1* and so forth, may be potential targets for HC regeneration.

Our organoid expansion experiments showed that *Gpm6b* could promote the proliferation of SCs in vitro. However, EdU^+^/SOX2^+^ proliferative SCs were not observed in vivo. At the stage of organoid proliferation culture, a variety of cytokines promoting cell growth were added to the medium, including EGF, IGF, FGF and CHIR. CHIR is a small molecule agonist of Wnt,^44^ and the presence of these cytokines makes it easier for cells to enter the cell cycle. The synergistic effect of these factors may be involved in the promotion of organoid formation by *Gpm6b*. However, the in vivo environment lacks the cofactors mentioned above. This explains, to a certain extent, the failure of *Gpm6b* to promote SC proliferation in vivo and suggests that *Gpm6b* might have a stronger effect on SC proliferation in the presence of other signals, such as Wnt. Thus, multiple signalling pathways and multiple genes may be required for cooperative regulation if proliferation is also desired in vivo.

In summary, we used AAV‐ie to up‐regulate *Gpm6b* in the cochlear SCs of newborn mice and discovered that *Gpm6b* overexpression led to a remarkable increase in HCs. Our study demonstrated for the first time the role of the *Gpm6b* gene in HC regeneration and its potential to participate in multiple signalling pathways to promote inner ear HC regeneration. At the same time we demonstrated that AAV was a powerful tool in hair cell regeneration applications. It is valuable for the future utilise of multiple genes and signalling pathways to jointly regulate inner ear HC regeneration via the recombined AAV.

## 4 MATERIALS AND METHODS

### 4.1 Animals

Lgr5‐EGFP^CreERT2^ and Rosa26‐tdTomato^loxP/+^ (The Jackson Laboratory, Stock No. 007914 and No. 008875, respectively) mice were bred in the animal room. Tamoxifen (Sigma, #T5648) was dissolved in corn oil and injected into P1 mice intraperitoneally (dose: 0.075 mg/g body weight). EdU (Beyotime, ST067‐50 mg) was dissolved in PBS and injected subcutaneously into P2–4 mice (dose: 0.05 mg/g body weight). All animal experiments were approved by the Institutional Animal Care and Use Committee of Southeast University.

### 4.2 HEK 293T cell culture

HEK 293T cells were taken out from liquid nitrogen and rapidly resuscitated in a 37°C water bath. After centrifuged at 1000 × *g* for 3 min, the cells were seeded in cell culture dishes (Greiner, #664160). The culture medium were DMEM (Gibco, #C11995500BT‐500 mL), 10% FBS (Vivacell, #C04001‐500), and 1% penicillin/streptomycin (Gibco, #15140122). When the cells grew to about 90% confluence, they were passaged according to the appropriate ratio (e.g., 1:3). For cell passage, 0.05% trypsin–EDTA (Gibco, #25200072) was added to lysis the cells after PBS washed. The cell morphology was observed under the microscope, when the cell boundary became significantly brighter, the 0.05% trypsin–EDTA was removed, then the digestion was terminated with culture medium, and the cells were gently blown off the bottom of the dish. The cells were collected into tubes and centrifuged at 1000 × *g* for 3 min. Next the cells were inoculated in cell culture dishes at an appropriate proportion for passage.

### 4.3 Organoid culture

The cochleae basal membranes of P1 WT mice were taken out and then they were digested into single cells with 0.25% trypsin. The digestion was terminated by adding trypsin inhibitor (Worthington, #59S11627), centrifuging at 2500 rpm, discarding the supernatant, and resuspending in DMEM/F12 (ThermoFisher, #11320033). Then the cells were resuspended in DMEM/F12 mixed with 35% Matrigel (Corning, #354230), and the cell resuspension was seeded in 24‐well dishes at 30 μL/well. The plates were then placed in a 37°C incubator for 30 min. When the Matrigel had solidified, proliferation medium was added (500 μL/well). The next day the AAV‐ie‐CAG‐mNeonGreen and AAV‐ie‐CAG‐*Gpm6b* viruses were added. The proliferation medium consisted of DMEM/F12, 2% B27 (ThermoFisher, #12587010), 1% N2 (ThermoFisher, #17502001), β‐FGF (10 ng/mL; #Peprotech, 100‐18C), EGF (20 ng/mL; life, #PHG0311), CHIR99021 (3 μM; Sigma‐Aldrich, #SML1046), valproic acid (1 mM; Sigma‐Aldrich, #P4543), 616452 (2 μm; Sigma‐Aldrich, #446859‐33‐2) and 0.1% ampicillin (Sangon Biotech, #A610028‐0025). After proliferation culture, the culture medium was replaced with differentiation medium, and the AAV‐ie‐CAG‐mNeonGreen and AAV‐ie‐CAG‐*Gpm6b* viruses were added. After 10 days of differentiation culture, the samples were collected. The components of the differentiation medium included DMEM/F12, 1% N2, 2% B27, CHIR99021 (3 μM), LY411575 (5 μM; Sigma‐Aldrich, #SML0506), and 0.1% ampicillin.

### 4.4 Virus packaging and purification

In the three‐plasmid packaging system, the target gene, viral capsid, and auxiliary plasmid were mixed with the transfection reagent PEI (yeasen; #40816ES02/03) according to the appropriate proportions, incubated at room temperature for 20 min, and then dropped into the culture dish of HEK 293 T cells at a density of about 90%. The culture medium was changed to 1% medium at 12 h, the supernatant was collected at 48 h and the cells were collected at 96 h. The components of the 1% medium were DMEM, 1% FBS and 1% penicillin/streptomycin. For virus purification, the method in the previous literature was used.^16^ The SYBR (Vazyme, #Q311) method was used for titre determination, and primers were designed from the WPRE region to determine the genomic titre of AAVs.

### 4.5 AAV injection through the round window membrane

Newborn mice were anaesthetised in ice. Under the microscope, a small incision was made between the ears and the neck of the mice with scissors. The fat and muscle were gently removed with tweezers to expose the round window of the cochlea. The AAV virus was injected into the mice cochlea through round window membrane via a glass micropipettes (Drummond, #5‐000‐1001‐X10). The incisions were sealed using tissue adhesive (3 M Vetbond, #1469SB), and the mice were placed on a 37°C heating pad to revive them before returning them to their mother's cage.

### 4.6 Immunofluorescence staining

Mouse cochlea and cells were fixed in 4% PFA (Beyotime, #P0099), and cochleae were decalcified by 0.5 M EDTA (Solarbio, #E1170). After the cochlea was cut under the microscope, it was blocked with 10% donkey serum (Solarbio, #017‐000‐121) for 1 h at room temperature. Primary antibodies against Myo7a (Proteus Biosciences, #25–6790, 1:1000 dilution) and Sox2 (Santa, #SC‐17320, 1:400 dilution) were incubated at 4°C overnight. Next day, they were washed with PBS and the corresponding secondary antibody was incubated at room temperature for 1 h. Finally, DAKO fluorescence mounting medium (DAKO, #S3023) was used to seal the samples. Images were taken under a laser confocal microscope.

### 4.7 Quantitative real‐time PCR and RNA sequencing

Total RNA from the tissue/organoids was extracted with Trizol (ThermoFisher, #15596018). Then the RNA was reverse‐transcribed into cDNA by a Reverse Transcription Kit (Vazyme, #R223‐01). The quantitative real‐time PCR (qPCR) was conducted with the SYBR (Vazyme, #Q712) on a Real‐Time PCR System (Bio‐Rad). The primer sequences are listed in Table 1. For RNA sequencing, all libraries were analysed through the bioanalyzer from Novogene for quality and concentration. Sequencing data was analysed using the NovoMagic Platform.

**TABLE 1:** The primer sequences were used in this study.

Gene	Forward sequence (5′–3′)	Reverse sequence (5′–3′)
Gpm6b	TGGGCTTACTTAAAGGATGCAAG	TTGAGTTGTTCTTTTGAGCGAGA
Lgr5	TCTTCACCTCCTACCTGGACCT	GGCGTAGTCTGCTATGTGGTGT
Cdc20	TTCGTGTTCGAGAGCGATTTG	ACCTTGGAACTAGATTTGCCAG
Gadd45a	CCGAAAGGATGGACACGGTG	TTATCGGGGTCTACGTTGAGC
Pmp22	CATCGCGGTGCTAGTGTTG	AAGGCGGATGTGGTACAGTTC
Stmn1	TCTGTCCCCGATTTCCCCC	AGCTGCTTCAAGACTTCCGC
Pou3f1	GCGAGCACTCGGACGAGG	CGCAGACGGCTTGGGACACT
Foxd3	CCCATCACGGACAGCCTCAG	TAGGCTGTTCTTGGGCTTGC
Fn1	ATGTGGACCCCTCCTGATAGT	GCCCAGTGATTTCAGCAAAGG
Col1a1	GCTCCTCTTAGGGGCCACT	CCACGTCTCACCATTGGGG
Id2	ATGAAAGCCTTCAGTCCGGTG	AGCAGACTCATCGGGTCGT
Lrp5	AAGGGTGCTGTGTACTGGAC	AGAAGAGAACCTTACGGGACG
Hipk2	TTTCTCCCCTCACACCCTTCA	CCAGTTGGAACTTGGCTCTACT
Sox9	AGTACCCGCATCTGCACAAC	ACGAAGGGTCTCTTCTCGCT
Cdh23	GGAGGATTACCTACGGCTCAA	GTGTGGATCAGCTCGGAGA
Espn	CCACAGGCTACCTCTCTTGC	AGCAGCCACTTCACCACATC
Gapdh	AGGTCGGTGTGAACGGATTTG	TGTAGACCATGTAGTTGAGGTCA
WPRE	GTCAGGCAACGTGGCGTGGTGTG	GGCGATGAGTTCCGCCGTGGC

### 4.8 Statistical analysis

All of the data were displayed as the mean with SEM, and statistical analyses were performed using GraphPad Prism9 software. For all experiments, *n* represents the number of replicates, and at least three individual experiments were conducted. Two‐tailed, unpaired Student's *t*‐tests were used to determine statistical significance when comparing two groups. In all cases, *p* < 0.05 was considered significant.

## AUTHOR CONTRIBUTIONS

R.C., F.Y. and J.Q. conceived and designed the experiments. Q.S., L.Z., T.C. and N.L. performed most of the experiments. X.G., Z.Z., Y.Z., J.L., X.Q. and Y.L. performed genotyping and cell culture. R.C., J.Q., F.T, B.G. and Q.S. discussed the data analysis, interpretation and presentation and wrote the manuscript with contributions from all authors.

## FUNDING INFORMATION

This work was supported by the National Key Research and Development Program of China (2021YFA1101300, 2021YFA1101800, 2020YFA0113600 and 2020YFA0112503), the STI2030‐Major Projects (2022ZD0205400), the National Natural Science Foundation of China (82030029, 81970882, 93149304, 82000984, 82071046 and 82371156), the China National Postdoctoral Program for Innovative Talents (BX20200082), the China Postdoctoral Science Foundation (2020M681468), the Science and Technology Department of Sichuan Province (2021YFS0371), the Natural Science Foundation from Jiangsu Province (BK20211168), the Shenzhen Science and Technology Program (JCYJ20210324125608022), the Open Research Fund of State Key Laboratory of Genetic Engineering, Fudan University (SKLGE‐2104), the Jiangsu Postdoctoral Research Funding Program (2021K156B) and the Fundamental Research Funds for the Central Universities.

## CONFLICT OF INTEREST STATEMENT

The authors declare that they have no competing interests.

## Data Availability

All data associated with this study are present in the paper.
